# Positive real-world effectiveness of tafamidis for delaying disease progression in transthyretin familial amyloid polyneuropathy

**DOI:** 10.1186/1750-1172-10-S1-P4

**Published:** 2015-11-02

**Authors:** Michelle Stewart, Denis Keohane, Sarah Short, Jose Alvir, Moh-Lim Ong, Rajiv Mundayat

**Affiliations:** 1 Pfizer Inc., New York, NY, USA

## Background

Tafamidis (Vyndaqel) was approved by the EMA in 2011 and is emerging as the standard of care for transthyretin familial amyloid polyneuropathy (TTR-FAP) in clinical settings. Efficacy was demonstrated in the clinical trials, yet little is known about its real-world effectiveness. A global disease registry, the Transthyretin Amyloidosis Outcomes Survey (THAOS), collects data on both treated and untreated patients from real-world settings. Ethics committee approval was obtained prior to patient enrolment.

## Objective

To demonstrate the real-world effectiveness of tafamidis.

## Methods

THAOS registry data were used to match 258 treated patients to untreated controls in a 1:4 non-randomized retrospective cohort study. Genetic mutation, birth region, and propensity scores derived from clinical status variables were used in matching. Descriptive statistics were calculated. Treatment effects were tested by repeated measures analyses with appropriate covariates (age, gender, disease duration, propensity score, and baseline values).

## Results

The matched sample was predominantly Val30Met (93%) with roughly equal gender ratio (52% male) and an average age of 41.4 years. Less disease progression was seen in the tafamidis treated group over 24 months on neurological and quality of life endpoints. The neurologic endpoints with statistically significance favoring tafamidis include the derived NIS-LL and the Neurologic Composite Score including sub-scores. The Norfolk TQoL Score was also statistically significant favoring tafamidis treatment. No significant differences were found for the modified BMI or the Karnofsky Performance Status Index.

## Conclusion

Tafamidis treatment resulted in less neurological progression. The results extend the efficacy observed in the clinical trials to real-world clinical settings.

